# Expression and significance of *SOX B1* genes in glioblastoma multiforme patients

**DOI:** 10.1111/jcmm.17120

**Published:** 2021-12-24

**Authors:** Cunyao Pan, Lanlan Liang, Zirou Wang, Baoyi Zhang, Qionglin Li, Yingrui Tian, Yijing Yu, Zhaoli Chen, Xinxing Wang, Hui Liu

**Affiliations:** ^1^ School of Public Health Lanzhou University Lanzhou China; ^2^ Tianjin Institute of Environmental and Operational Medicine Tianjin China

**Keywords:** bioinformatics analysis, glioblastoma, overall survival, *SOX B1* family members

## Abstract

The overall survival of glioblastoma multiforme (GBM) patients remains poor. To improve patient outcomes, effective diagnostic and prognostic biomarkers for GBM are needed. In this study, we first applied bioinformatic analyses to identify biomarkers for GBM, focusing on *SOX* (sex‐determining region on the Y chromosome (SRY)‐related high mobility group (HMG) box) *B1* family members. The ONCOMINE, GEPIA, LinkedOmics and CCLE databases were used to assess mRNA expression levels of the *SOX B1* family members in different cancers and normal tissue. Further bioinformatic analysis was performed using the ONCOMINE database in combination with the LinkedOmics data set to identify the prognostic value of *SOX B1* family members for GBM. We found mRNA expression levels of all tested *SOX B1* genes were significantly increased in GBM. In the LinkedOmics database, increased expression of *SOX3* indicated a better overall survival. In GEPIA databases, increased expression of all *SOX B1* family members suggested an improved overall survival, but none of them were statistically different. Then, Transwell assays and wound healing were employed to evaluate the motility and invasive captivity of *U251* cells when silencing *SOX2* and *SOX3*. We found exogenous inhibition of *SOX2* appeared to reduce the migration and invasion of *U251* cells in vitro. Collectively, our research suggested that *SOX2* might serve as a cancer‐promoting gene to identify high‐risk GBM patients, and *SOX3* had the potential to be a prognostic biomarker for GBM patients.

## 1 INTRODUCTION

Glioblastoma multiforme (GBM) is the most common, malignant and high‐grade brain tumour.^1^, ^2^ The WHO classification system divides glioma into 4 subtypes and GBM as grade 4 glial tumour has the worst prognosis.^3^ The intra‐ and intertumoral genetic and epigenetic heterogeneity observed in GBM highlights the complexity of cancer.^4^ The median survival of GBM patients is around 12–15 months, even with the use of surgery, radiotherapy, chemotherapy and immunotherapy.^5^ To date, no specific biomarkers that offer improvements to GBM patient survival have been found. Therefore, it is necessary to identify strategies and targets for early diagnosis and prognosis of GBM.

*SOX (*sex‐determining region on the Y chromosome‐related high mobility group box) *B1* genes consist of *SOX1*, *SOX2* and *SOX3*, sharing a high degree of sequence similarity, both within and outside the high mobility group box.^6^ Previous studies have discovered that *SOX B1* members are widely expressed in neural tissue, embryonic stem cells and testes.^7^ They are involved in various physiological processes, such as maintaining embryonic stem cell function, the occurrence of neural tissues, controlling male sex determination and maintaining neural stem cells.^8^, ^9^ In addition, dysregulated expression of *SOX B1* family members affect the occurrence and prognosis of various cancers. Accumulating evidence suggests that *SOX B1* genes have both antiproliferative and prosurvival effects, depending on cancer. On the one hand, *SOX B1* members have been shown to be tumour suppressors in nasopharyngeal carcinoma,^10^ ovarian cancer^11^ and metrocarcinoma.^12^, ^13^ On the other hand, *SOX B1* genes have been shown to play an oncogenic role in breast cancer,^14^, ^15^ squamous cell carcinoma,^16^ hepatocellular carcinoma,^17^ osteosarcoma^18^, ^19^ and brain cancer.^20^, ^21^ Consequently, a greater understanding of their role in various cancers is needed.

In recent years, proteomics, transcriptomics and high‐throughput sequencing technologies have developed rapidly, generating a large amount of genomics data in the field of cancer research. Although the relationship between the *SOX B1* genes and many cancers has been partly reported, no study has fully summarized their role via bioinformatics. Based on multiple published databases, we analysed the expression of *SOX B1* family members to determine their expression levels in various cancers, with focus on their diagnostic and prognostic value in GBM.

## 2 MATERIALS AND METHODS

### 2.1 ONCOMINE database

ONCOMINE is the largest and most comprehensive gene chip database and data extraction platform **(** https://www.oncomine.org/resource/login.html), containing 715 databases and 86,733 samples that can be used to compare differences between cancer and normal tissue.^22^ It was used to compare the transcription levels of *SOX B1* members in different cancers. The mRNA expression levels of the *SOX B1* genes in clinical cancer specimens were compared with levels in normal specimens (controls), using a Student's t‐test to generate a *p*‐value. The *p*‐value cut‐off was defined as 0.05.

### 2.2 GEPIA database

GEPIA database (Gene Expression Profiling Interactive Analysis) (http://gepia.cancer‐pku.cn/) is an online database developed by Peking University that performs a dynamic analysis of gene expression spectrum data. The expression of genes in different tumours was analysed in combination with TCGA (The Cancer Genome Atlas) and GTEx databases (Genotype‐Tissue Expression). *SOX B1* gene survival analysis was carried out by the method of total survival rate and disease‐free survival rate.

### 2.3 LinkedOmics data set

LinkedOmics data set (http://linkedomics.org/login.php) is an online tool for analysing TCGA databases. This data set was used to examine the relationship between mRNA levels of *SOX B1* genes and the overall survival of GBM patients.

### 2.4 Cancer Cell Line Encyclopaedia database

The Cancer Cell Line Encyclopaedia (CCLE) database (www.broadinstitute.org/ccle/home) is a project to develop the integrated computational analysis of human cancer models and molecular spectrum of nearly 1,000 human cancer cell lines used in global drug research and development.^23^ The *SOX B1* members’ expression is affirmed via the CCLE database.

### 2.5 Cell culture

*U251* was purchased from the Guangzhou Institute of Biomedicine and Health, Chinese Academy of Sciences. *U251* cells were cultured in DMEM (high glucose) containing 10*%* foetal bovine serum and 1% penicillin/streptomycin in a T75 culture flask and in logarithmic growth phase were used for further experiments.

### 2.6 Lipofection transfection

*U251* cells were transfected with Lipofectamine2000 Reagent (Invitrogen) according to the manufacturer's instructions. *U251* cells were seeded in 6‐well plates and transfected with scramble siRNA, siRNA purchased from Suzhou Jima Gene Co, Ltd. siRNA‐SOX2 (the sense primer 5‐CCAUGGGUUCGGUGGUCAATT‐3 CCAU and antisense primer 5‐UUGACCACCGAACCCAUGGTT‐3) and siRNA‐SOX3 (the sense primer 5‐CUCAGAGCUACAUGAACGUTT and antisense primer 5‐ACGUUCAUGUAGCUCUGAGTT‐3) when reaching 80%–90% density. The Opti‐MEM medium was used to dilute lipofectamine reagent (10 μl:150 μl)and siRNA (14 μl:175 μl). After mixing, the solution was stood for 5 min. Then, 260 μl mixture was added in Opti‐MEM medium, and the final overall volume was 2 ml. The old culture medium was sucked and washed with PBS twice. Finally, the mixture was placed into the 6‐well plates, gently blended and then placed into a 37 °C 5% CO_2_ incubator for further culture. After 4 h, the mixture was removed and replaced with medium containing 10% serum but no antibiotics.

### 2.7 Wound‐healing migration assay

Before cell seeding, a vertical line and 5 horizontal lines were drawn at 0.5‐cm intervals on the back of a transparent 6‐well plate to allow localization of cells during image acquisition. Cell culture and transfection were performed as described above. When cell density reached a 100 percent confluent monolayer, scratches were made with a gun head (200μL) perpendicular to the horizontal line on the back of the 6‐well plate. The cells were washed with PBS 3 times to remove the scratched cells, and 2 ml of serum‐free medium were added to each well. Pictures were taken with a microscope before the plate was placed in an incubator for further culture. After 48 h, cells were washed with PBS twice and photographed under microscopy observation.

### 2.8 Transwell invasion assays

Twenty‐four hours after transfection, 1 × 10^5^ cells in serum‐free DMEM were plated in the upper chamber (the total volume was 200 ml) while 600 μl of the full medium was placed in the lower chamber. Small chambers were then removed from the 24‐well plate 72 h after incubation. Nonadherent cells were washed away 3 times with PBS and nonmigrated cells on top of the membrane were removed with a cotton swab. Invading cells were fixed with 4% formaldehyde for 30 min and stained with 0.1% haematoxylin for 30 min. After washing with PBS twice, cells were mounted under microscope observation.

### 2.9 Quantitative real‐time polymerase chain reaction

For RNA extraction, the Trizol reagent (Life Technologies) was used in accordance with the manufacturer's instructions. Real‐time quantitative *PCR* (*RT*‐*qPCR*) was performed with SYBR Green Real‐time PCR Master Mixes (Thermo Fisher Scientific, USA), and β‐actin served as an internal control. Our results were analysed using $2^{‐\Delta \Delta C_{T}}$ method. All primer sequences are listed in detail in Table S1.

### 2.10 Statistical analysis

Statistical analyses were carried out using *Graph Pad* Prism 8. The data are presented as the mean ±SD. Statistical testing was performed using an unpaired t‐test for two group comparisons, One‐way ANOVA with a post hoc test was applied for multigroup comparisons. *p* < 0.05 was considered to indicate statistical significance.

## 3 RESULTS

### 3.1 Assessing expression levels of *SOX B1* genes in different cancers using the ONCOMINE database

**FIGURE 1:**
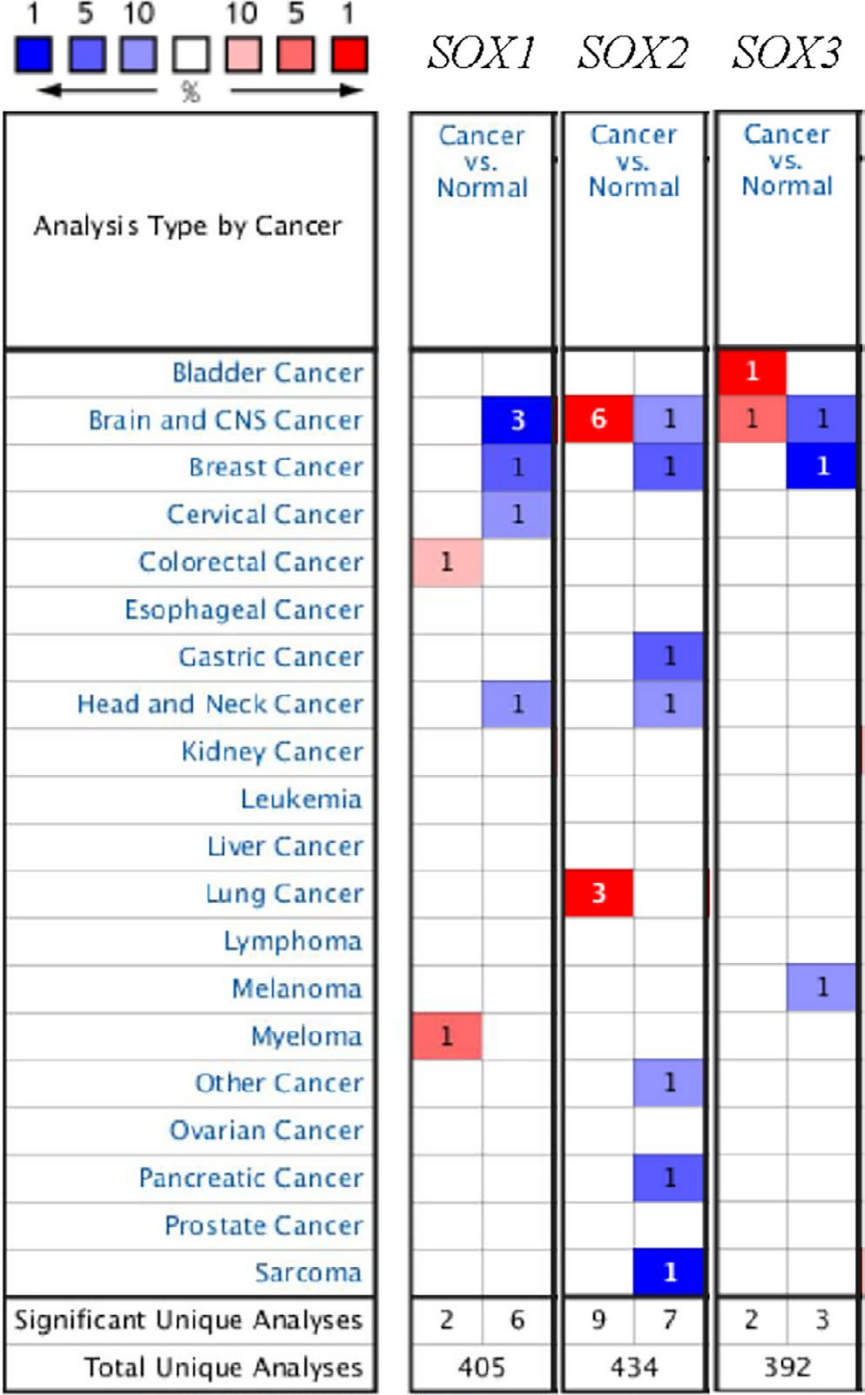
The transcription levels of *SOX B1* subgroups in different types of cancers (ONCOMINE)

We compared the expression levels (mRNA) of *SOX B1* family members (SOX1, SOX2 and SOX3) in various cancers and normal tissue using the ONCOMINE database. *SOX B1* genes were found to be differentially expressed in different cancers (Figure 1). Collectively, *SOX B1* genes have been most studied in the brain and central nervous system. In the ONCOMINE database, there are 12 databases that record the mRNA levels of the *SOX B1* family in the brain and central nervous system. Six databases indicated that *SOX2* was overexpressed in the brain and the central nervous system, making it the most studied *SOX B1* family gene here.

### 3.2 Assessing expression levels of the *SOX B1* genes in different cancers using the GEPIA database

**FIGURE 2:**
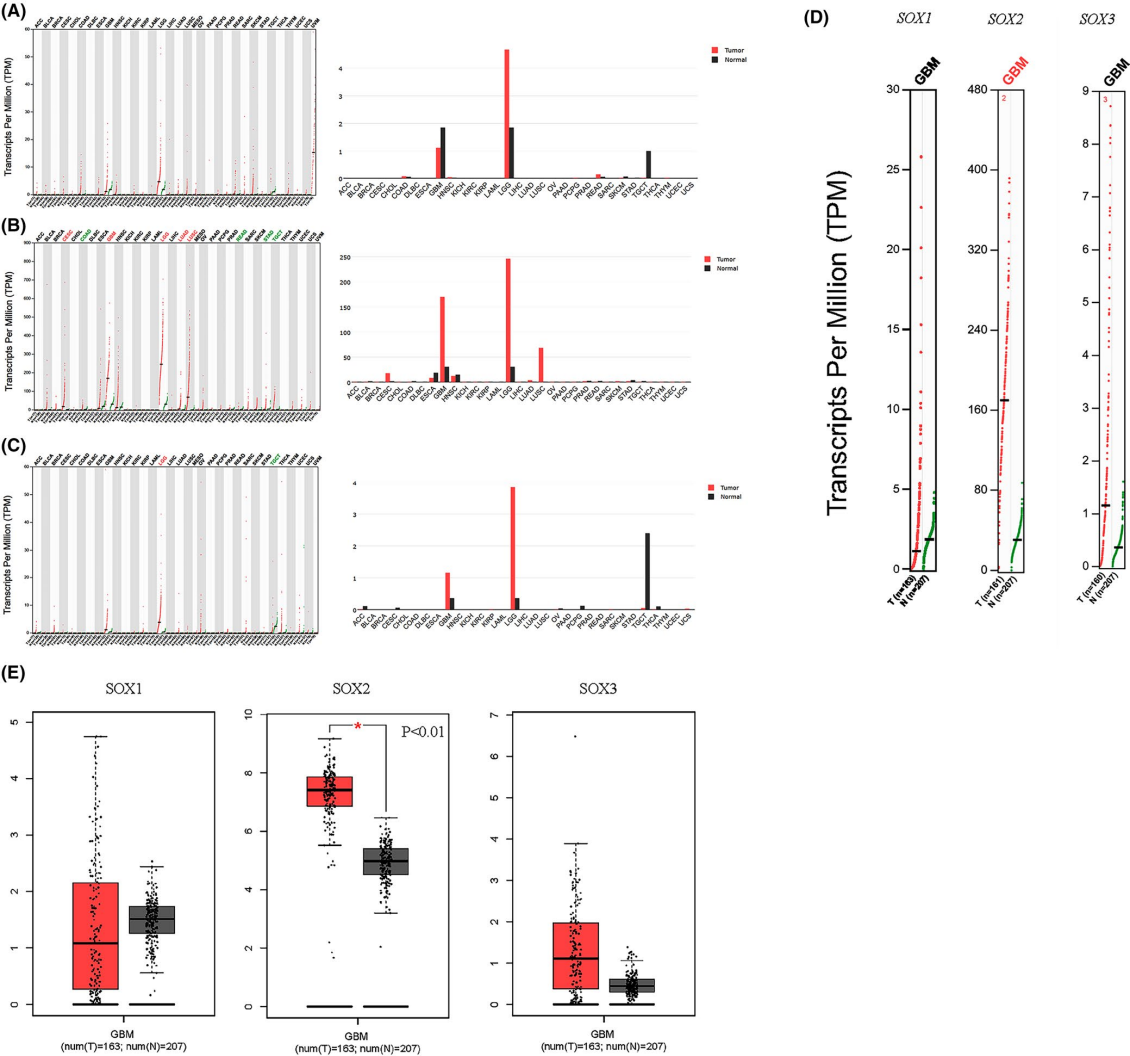
The expression of SOX *B1* subgroups in different types of cancers (GEPIA). (A) The expression of *SOX1* in pan‐cancer. (B) The expression of *SOX2* in pan‐cancer. (C) The expression of *SOX3* in pan‐cancer. (D–E) The expression of *SOX B1* subgroups in GBM

We compared the mRNA levels of *SOX B1* family members in pan‐cancer using the GEPIA database. The results suggested that *SOX B1* gene expression was higher in GBM and low‐grade glioma (LGG) patients than in normal tissue samples. Because LGG patients have always been characterized by a relatively good prognosis,^24^, ^25^ we focused on the expression levels of *SOX B1* genes in GBM. The results showed that GBM patients had higher *SOX2* expression levels than healthy patients (*p* < 0.01) (Figure 2A–2E).

### 3.3 Assessing expression levels of the *SOX B1* genes in different cancers using the CCLE database

**FIGURE 3:**
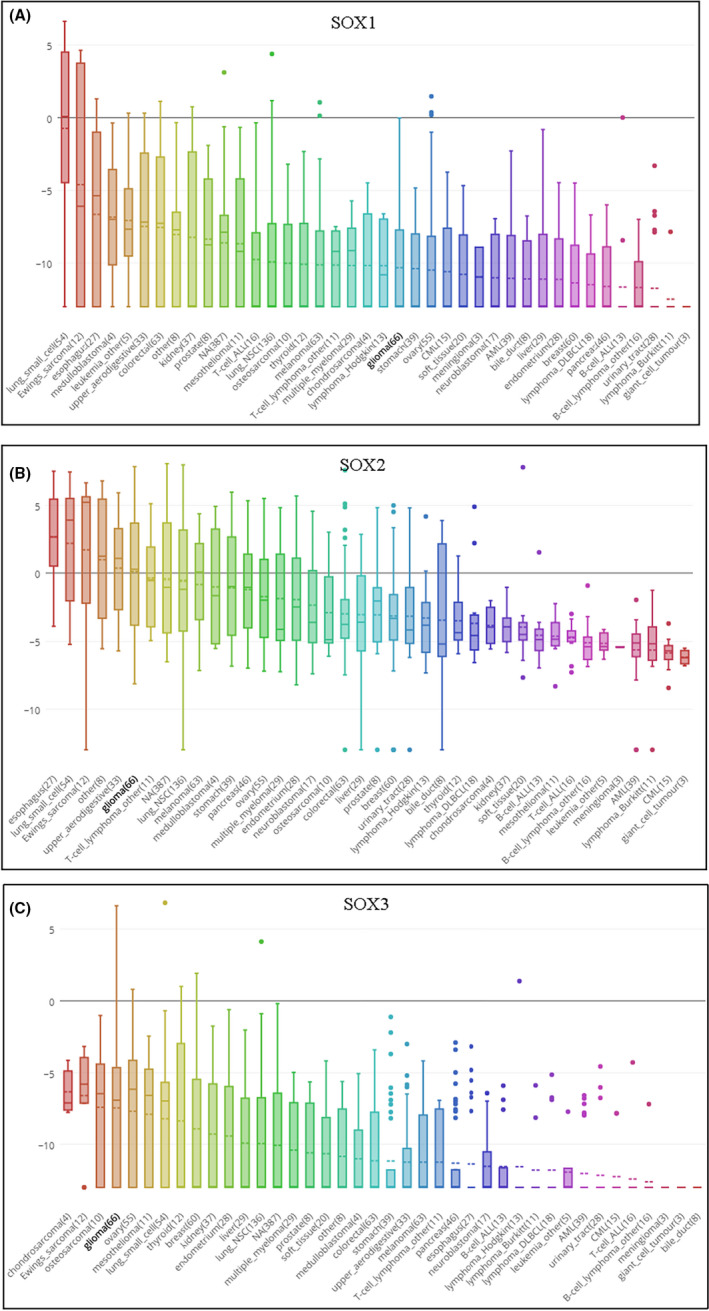
The Expression of *SOX B1* subgroups in GBM Cell Lines (analysed by CCLE). (A) The expression of *SOX1* in cancer cell lines. (B) The expression of *SOX2* in cancer cell lines. (C) The expression of *SOX3* in cancer cell lines

We compared the expression levels of SOX B1 genes using the CCLE database. Consistent with the previous findings (above), the mRNA levels of *SOX2* and *SOX3* were higher in glioma cell lines compared to other cancer types (Figure 3A–3C).

### 3.4 Assessing changes in *SOX B1* family member expression in GBM patients

We compared the mRNA expression levels of *SOX B1* genes between GBM and normal tissue using the ONCOMINE database. In all statistically significant data sets, all *SOX B1* genes were upregulated, to varying degrees, compared to normal tissue (Table 1). In Murat Brain's data set,^26^ *SOX1* was overexpressed in GBM samples with a fold change (FC) of 1.122 (Table 1). *SOX2* was overexpressed in four data sets (Table 1). SOX2 was overexpressed in Sun Brain's data set^27^ (FC = 2.515), Shai Brain's data set^28^ (FC = 1.983), Murat Brain's data set^29^ (FC = 1.496) and TCGA Brain's data set (FC = 2.401). In Murat Brain data set,^29^ S*OX3* was overexpressed with an FC of 1.184 (Table 1).

**TABLE 1:** Observed significant changes in *SOX B1* family member expression (mRNA) between glioblastoma and normal samples

Gene ID	Fold Change	*p*‐Value	t‐Test	Reference
SOX1	1.122	0.007	4.105	Murat Brain^26^
SOX2	2.515	2.67E‐18	10.791	Sun Brain^27^
	1.983	1.61E‐5	5.392	Shai Brain^28^
	1.496	5.43E‐6	9.680	Murat Brain^26^
	2.401	0.014	3.249	TCGA Brain
SOX3	1.184	0.022	2.729	Murat Brain^26^

### 3.5 Prognostic analysis of *SOX B1* subgroups

**FIGURE 4:**
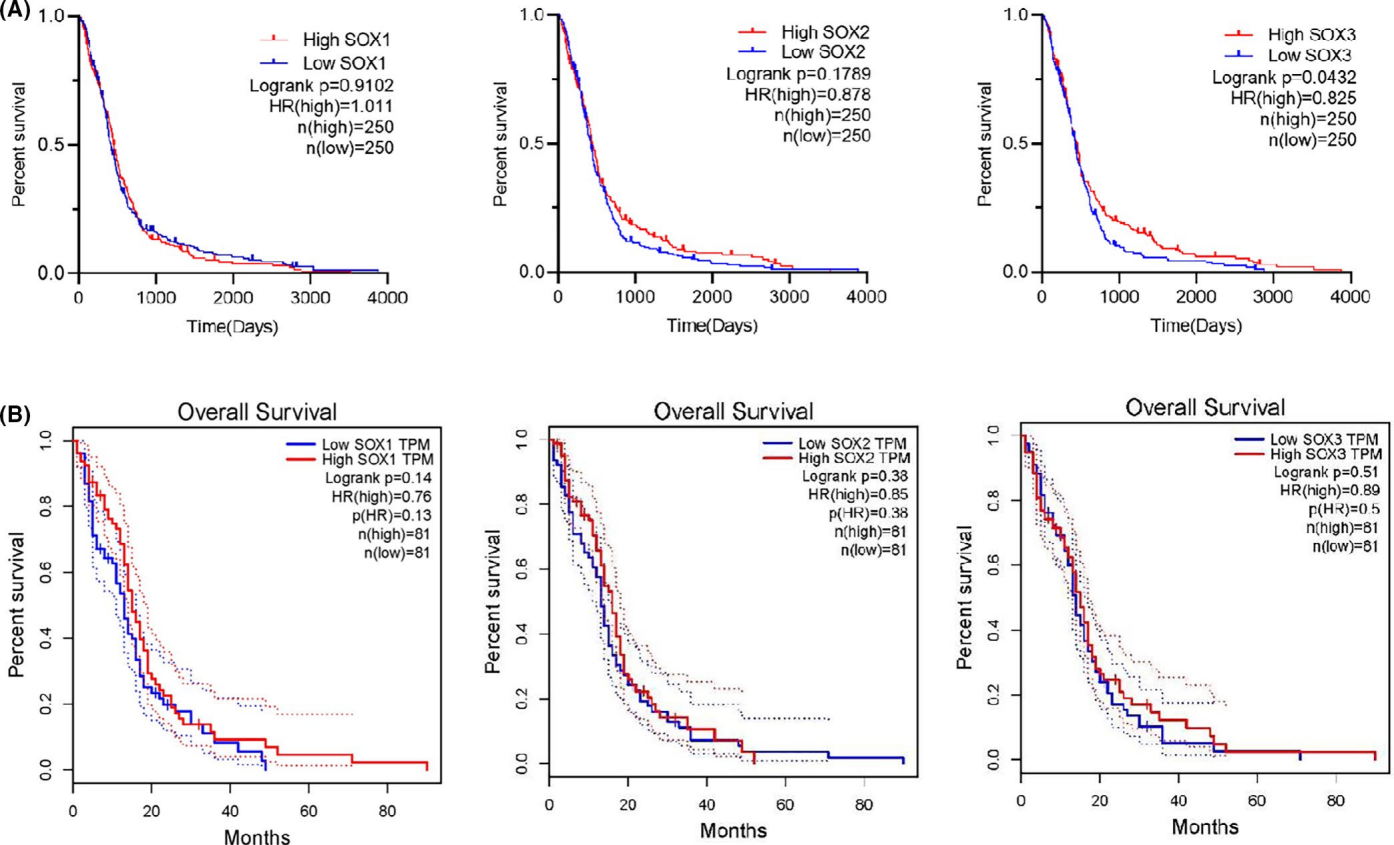
The prognostic value of mRNA Level of *SOX B1* factors in GBM (LinkedOmics and GEPIA). (A) The prognostic value of mRNA level of *SOX* factors in GBM patients, analysed by LinkedOmics. (B) The prognostic value of mRNA level of *SOX* factors in GBM patients, analysed by GEPIA

We conducted a prognostic analysis for *SOX B1* genes by using LinkedOmics and GEPIA databases. The data indicated that decreased *SOX1* expression levels predicted better overall survival in GBM, while decreased *SOX2* indicated poor overall survival, but neither result was statistically significant. To our surprise, increased *SOX3* showed better overall survival (Logrank *p* = 0.0432) (Figure 4A). The survival rate of high *SOX3* patients is much higher than low *SOX3* patients (HR = 0.825). In the GEPIA database, increased *SOX B1* expression indicated a better overall survival, but the results were not statistically significant (Figure 4B).

### 3.6 Co‐expression and correlation analysis of *SOX B1* family members

**FIGURE 5:**
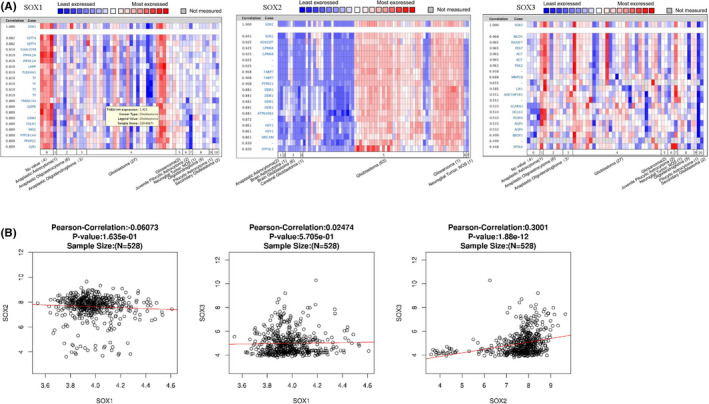
Co‐expressed genes of *SOX B1*, and the correction between SOX in (ONCOMINE and LinkedOmics). (A) Co‐expressed genes of *SOX B1*in GBM, analysed by ONCOMINE. (B) The correction between *SOX B1* in GBM, analysed by LinkedOmics

Co‐expression of *SOX1* was analysed in Bredel Brain2′ data set,^30^ and we found *SOX1* was positively corrected with *SEPT4*, *KIAA1598*, *PIP4K2A*, *LHPP*, *PLEKHH1*, *TF*, *TMEM144*, *QDPR*, *GRM3*, *FOLH1*, *MOG*, *PPP1R14A*, *PPAP2C* and *GJB1*(Figure 5A). S*OX2* was analysed in Schulte Brain's data set,^31^ and *SOX2* was positively corrected with *SOX2OT*, *GPM6B*, *FABP7*, *PTPRZ1*, *DDR1*, *ATP6V0E2*, *HEY1*, *NRCAM* and *DPYSL5* (Figure 5B). *SOX3* was analysed in Bredel Brain2’s data set,^30^ and *SOX3* was positively corrected with *BICD1*, *RASSF7*, *PEX7*, *AGT*, *TNK2* (Figure 5C).

We analysed the correlation among *SOX1*, *SOX2*, and *SOX3* via the LinkedOmics database. We found there was no significant correction between *SOX1* and *SOX2* or also *SOX1* and *SOX3*. However, *SOX2* and *SOX3* expression was positively correlated. (R = 0.3001, *p* < 0.05) (Figure 5D).

### 3.7 Effects of SOX2 and SOX3 downregulation on migration ability and invasion abilities of U251 cells

**FIGURE 6:**
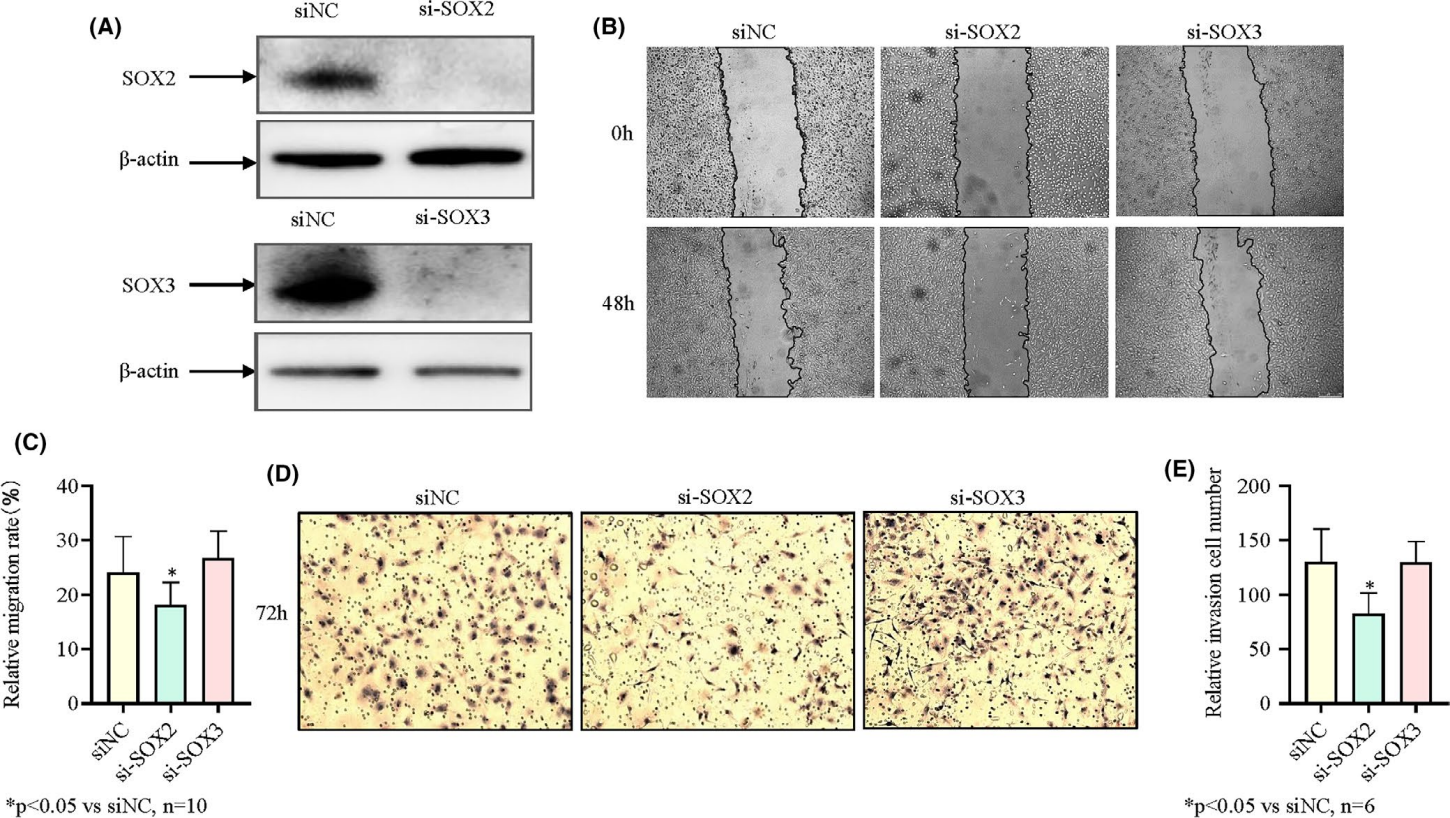
Effects of SOX2 and SOX3 downregulation on migration and invasion abilities of *U251* cells. (A) The *U251*cell extracts were subjected to western blotting to determine the SOX2 and SOX3 levels after transfected siSOX2 and siSOX3. β‐Actin was used as a protein loading control. (B) The experimental result of the scratch test after transfection (×100). (C) Quantitative analysis of (B). (D) Transwell chamber invasion assay after transfection (×200). (E) Quantitative analysis of (D)

The silencing effect was confirmed by western blotting (Figure S1 and Figure 6A). The downregulation of SOX2 and SOX3 to influence tumour cell migration was evaluated using wound‐healing migration assay. Figure 6B and C showed representative microscopy images at 0h and 48h, together with the relative quantitative analysis of wound‐closure rate. The wound‐healing assay showed that the wound‐healing rate of the control group (transfected with scramble RNA) was 24.10. Downregulation of SOX2 significantly decreased the wound‐healing rate in *U251* cells at 48 h (24.10 ± 6.59, siNC vs 18.11 ± 4.16, siSOX2; *p *< 0.05). Downregulation of SOX3 increased the wound‐healing rate in *U251* cells at 48 h (24.10 ± 6.59, siNC vs 26.75 ± 4.93, siSOX3; *p *> 0.05). These results suggested that downregulation of SOX2 negatively influenced the wound closure rate of *U251* cells. Downregulation of SOX3 showed an opposite effect, but was not statistically significant.

The downregulation of SOX2 and SOX3 to influence tumour cell invasion was examined with Transwell invasion assays. Figure 6D and 6E show representative microscopy images at 72 h, together with quantification analysis of invasion ability of *U251* cells measured using Transwell invasion assay. The results of the Transwell invasion assay revealed the numbers of invasive cells about 130 in the control group. Downregulation of SOX2 significantly decreased the number of invasive cells at 72 h (130.17 ± 30.18, siNC vs 82.17 ± 19.45, siSOX2; *p *< 0.05), downregulation of SOX3 has no significant effect on *U251* cell invasion. (130.17 ± 30.18, siNC vs 129.50 ± 19.48, siSOX3; *p *> 0.05). These results suggested that the downregulation of SOX2 negatively influenced the invasion ability while SOX3 downregulation had no effect on the invasion of *U251* cells.

### 3.8 Downregulation of SOX2 affected the expression of invasion and apoptosis‐related gene.

**FIGURE 7:**
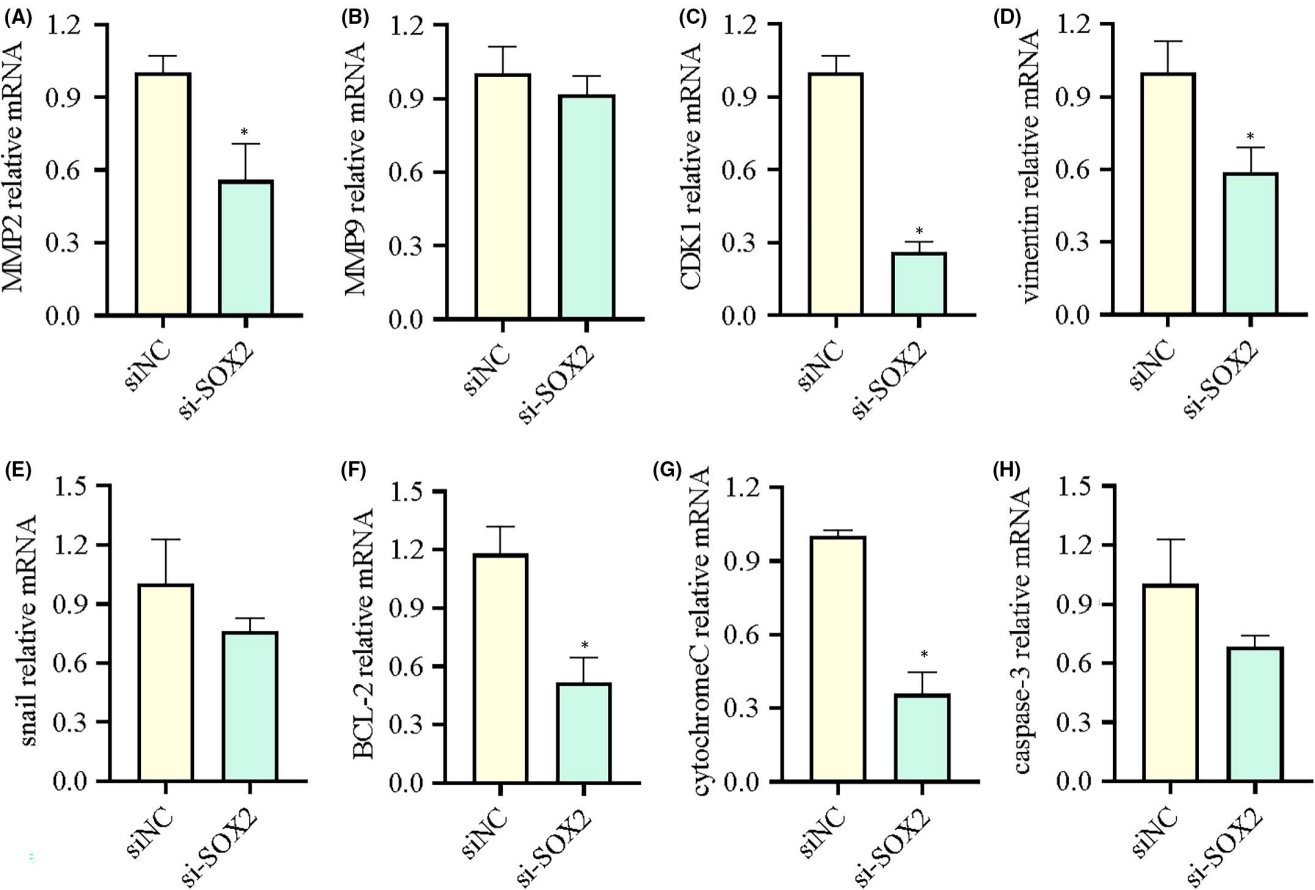
Downregulation of SOX2 affected the expression of invasion and apoptosis‐related gene. (A) Effects of SOX2 downregulation on the mRNA expression of *MMP2*. (B) Effects of SOX2 downregulation on the mRNA expression of *MMP9*. (C) Effects of SOX2 downregulation on the mRNA expression of *CDK1*. (D) Effects of SOX2 downregulation on the mRNA expression of *vimentin*. (E) Effects of SOX2 downregulation on the mRNA expression of *snail*. (F) Effects of SOX2 downregulation on the mRNA expression of *BCL*‐*2*. (G) Effects of SOX2 downregulation on the mRNA expression of *cytochrome C*. (H) Effects of SOX2 downregulation on the mRNA expression of *caspase*‐*3*. The symbol (*) indicates a significant change in comparison between marked groups (*p* < 0.05)

According to the aforementioned experimental results, the effect of silenced SOX2 on expression of invasion and apoptosis‐related gene including *MMP2*, *MMP*9, *CDK1*, *vimentin*, *cytochrome C*, *BCL*‐*2*, *snail* and *caspase*‐*3* was examined via RT‐qPCR (Figure 7A–7H). The results showed that downregulation of SOX2 downregulated the mRNA expression levels of *MMP2*, *CDK1*, *vimentin*, *BCL*‐*2* and *cytochrome C*.

## 4 DISCUSSION

The prognosis of gliomas is closely related to its histological type and tumour grade. Therefore, early and precise diagnosis of GBM is a key aspect for prognosis. Lei et al.^4^ indicated founding immunohistochemical marker of SRSF1 can be a promising diagnostic method for GBM. Stella M et al.^32^ suggested circHIPK3 and circSMARCA5 could be good diagnostic biomarkers for GBM. We found that *SOX B1* family members are differentially expressed in different cancers and that they could be a useful marker in the diagnosis and prognosis of GBM patients. By comparing the transcription levels of *SOX B1* in pan‐cancer through ONCOMINE and GEPIA data sets, *SOX2* was overexpressed in GBM and LGG and *SOX3* had elevated expression in LGG. By using the CCLE data set, we further confirmed that *SOX B1* genes were overexpressed in gliomas. Since the prognosis of LGG patients is relatively good, we focused on analysing the relationship between *SOX B1* genes and GBM. In 2007, Schmitz M et al. found that *SOX2* expression levels (mRNA and protein) were increased in human brain tumour biopsies.^33^ *SOX2* plays a vital role in the carcinogenesis and maintenance of GBM stem cells.^34^, ^35^ Subsequently, an increasing number of studies have shown that overexpression of *SOX2* is closely related to tumour invasiveness and poor prognosis.^36^, ^37^, ^38^ Silenced *SOX2* can inhibit proliferation and induce loss of tumorigenicity in GBM tumour‐initiating cells in immunosuppressant mice,^39^ and knockdown studies of *SOX2* reduced cellular proliferation and colony formation in a GBM cell line.^40^, ^41^ Our results showed SOX2 silencing significantly decreased proliferation of GBM cells, so SOX2 overexpression may contribute to tumour progression of GBM.

Furthermore, we analysed the prognostic value of *SOX B1* mRNA levels in GBM patients by using LinkedOmics and GEPIA databases. To our surprise, increased expression of *SOX2* had no influence on the prognosis of GBM patients. And, overexpressed *SOX3* indicated better overall survival in GBM patients (Logrank *p* = 0.0432, HR high = 0.825), suggesting that *SOX3* is an antioncogene. In contrast, previous studies have shown that overexpression of *SOX3* is associated with poor overall survival in gastric cancer,^42^ breast cancer^43^ and adult de novo acute myeloid leukaemia.^44^ Lu S et al.^45^ indicated that overexpression of *SOX3* predicts a poor outcome in GBM patients; and, Sa JK et al.^46^ found *SOX3* is associated with tumour invasiveness, malignancy and poor prognosis in GBM patients. Vicentic et al.^47^ found *SOX3* can accelerate the malignant behaviour of GBM cells. Hence, the definite role of *SOX3* in GBM was unclear. Finally, we conducted gene co‐expression analysis using the ONCOMINE database. The results indicated that *SOX B1* members were closely related to many different genes. However, *SOX1*, *SOX2* and *SOX3* did not share any co‐expressed genes. By using the LinkedOmics database, we found SOX2 and SOX3 expression was positively related.

In order to elucidate whether the expression of *SOX2* and *SOX3* affected the proliferation and invasion of glioma cells, we downregulated the expression of SOX2 and SOX3 to observe the changes of migration and invasion ability of *U251* cells. Wound‐healing migration assay and Transwell invasion assays have shown that SOX2 knockdown could inhibit *U251* cells migration and invasion, which were consistent with those of Luo et al.^48^ However, *SOX3* exerted little effect on cell migration and invasion. We further discovered that the mRNA of *MMP2*, *CDK1* and *vimentin* were significantly decreased after SOX2 downregulation in *U251* cells, suggesting that *MMP2*, *CDK1* and *vimentin* were associated with cell migration and invasion. Durinck et al.^49^ demonstrated that CDK1 played an important role during migration and invasion of cells. Upregulation of CDK1 promoted oncogenesis and progression of human gliomas, whereas downregulation of CDK1 and CDK2 expression inhibited the migration and invasion of human gliomas.^50^ As a mesenchymal marker, the downregulation of vimentin inhibited the migration and invasion ability of glioma cells. *Bcl*‐*2* is an anti‐apoptotic protein. Thus, inhibition of *Bcl*‐*2* expression may promote apoptosis. Decreased *SOX2* may contribute to apoptosis increase and reducing migration and invasion by inhibiting *Bcl*‐*2*.

## 5 CONCLUSION

In conclusion, we investigated the expression and prognostic value of *SOX B1* genes in GBM. We concluded that the expression of *SOX1*, *SOX2* and *SOX3* in GBM might result in tumorigenesis. Overexpressed *SOX2* could serve as a biomarker to identify high‐risk GBM patients. Moreover, *SOX2* may enhance the migratory and invasive capacity of glioma cells. Furthermore, *SOX3* may serve as a prognostic biomarker set for GBM patients. Hence, *SOX2* may serve as a potential therapeutic target in GBM patients, and more experiments are needed to clearly identify the specific mechanism of GBM formation pathway involved in *SOX2* and *SOX3*.

## CONFLICT OF INTEREST

The authors declare no conflict of interest.

## AUTHOR CONTRIBUTIONS

**Cunyao Pan:** Conceptualization (equal); Software (equal); Writing – original draft (equal). **Lanlan Liang:** Formal analysis (equal); Methodology (equal). **Zirou Wang:** Resources (equal). **Baoyi Zhang:** Methodology (equal). **Qionglin Li:** Software (equal). **Yingrui Tian:** Investigation (equal). **Yijing Yu:** Data curation (equal). **Zhaoli Chen:** Conceptualization (equal); Writing – review & editing (equal). **Xinxing Wang:** Conceptualization (supporting). **Hui Liu:** Conceptualization (lead); Project administration (lead).

## Supporting information

Supplementary Material

Supplementary Material

## Data Availability

All data, models and code generated or used during the study appear in the submitted article.
