# Supplementary material for: Expression and significance of SOX B1 genes in glioblastoma multiforme patients
Source: J Cell Mol Med. 2021 Dec 24;26(3):789–99. doi: 10.1111/jcmm.17120 (PMC8817144; doi:10.1111/jcmm.17120)
Supplement: Supplementary file 1 — Supplementary Material [file JCMM-26-789-s001.docx]

**Supplemental Information**

**Antibodies and WB assays**


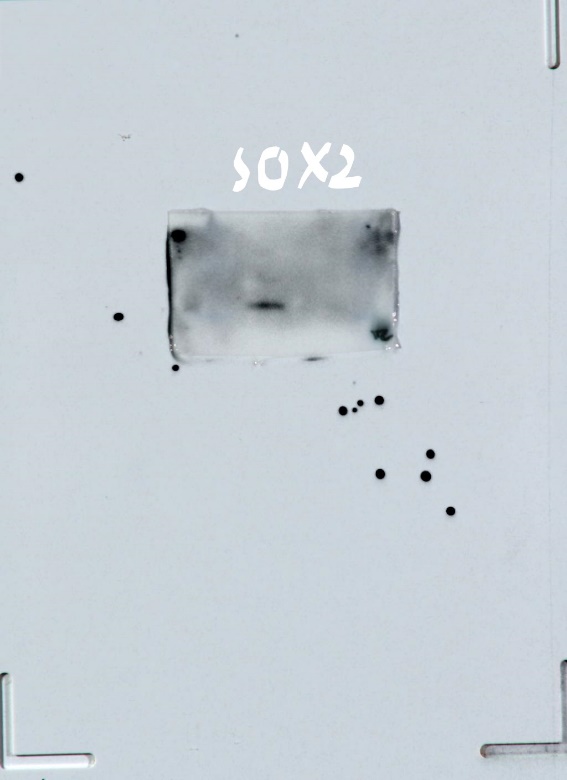

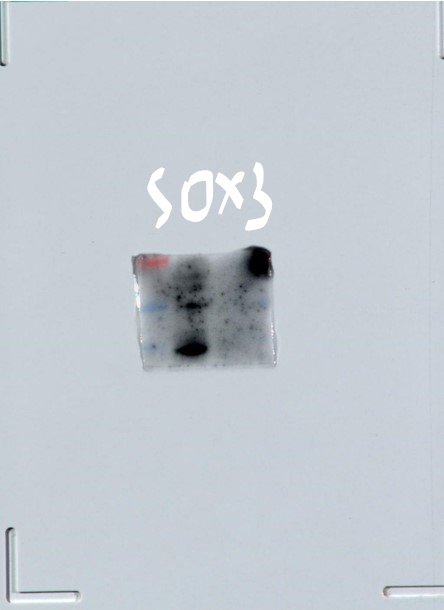
Antibodies specific for SOX2, and SOX3 were purchased from Abcam Technology (Cambridge, MA, USA). SOX2 and SOX3 expression were silenced via using siRNA in *U251* cells and were confirmed by WB assays as indicated below (Figure S1):

**Quantitative Real-Time PCR (qPCR)**

The primer sequences are shown as follow. Table S1

|  | Forward | reverse |
| --- | --- | --- |
| MMP9 | CAGGTGTGGGTGTACACAGG | CCTTCACGTCGAACCTCCAG |
| MMP2 | GAGTGCATGAACCAACCAGC | TGTTCAGGTATTGCATGTGCT |
| CDK1 | CCCTTTAGCGCGGATCTACC | CATGGCTACCACTTGACCTGT |
| vimentin | GGGACCTCTACGAGGAGGAG | TCCTCCTGCAATTTCTCCCG |
| BCL-2 | GGATAACGGAGGCTGGGATG | TGACTTCACTTGTGGCCCAG |
| caspase-3 | GGCGGTTGTAGAAGAGTTTCG | TCACGGCCTGGGATTTCAAG |
| Bax | AAACTGGTGCTCAAGGCCC | AAAGTAGGAGAGGAGGCCGT |
| Cytochrome C | CGCCAATAAGAACAAAGGCATCA | TAAGGCAGTGGCCAATTATTACTC |
| actin | GCCGCCAGCTCACCAT | TCGTCGCCCACATAGGAATC |
