# Supplementary material for: Expression and significance of SOX B1 genes in glioblastoma multiforme patients
Source: J Cell Mol Med. 2021 Dec 24;26(3):789–99. doi: 10.1111/jcmm.17120 (PMC8817144; doi:10.1111/jcmm.17120)
Supplement: Supplementary file 2 — Supplementary Material [file JCMM-26-789-s002.docx]

**Supplementary Table.** All primer sequences are listed in detail in Table S1.
